# Patients With Deep Ovarian Suppression Following GnRH Agonist Long Protocol May Benefit From a Modified GnRH Antagonist Protocol: A Retrospective Cohort Study

**DOI:** 10.3389/fendo.2021.618580

**Published:** 2021-07-13

**Authors:** Shan Liu, Minghui Liu, Lingxiu Li, Huanhuan Li, Danni Qu, Haiying Ren, Hui Su, Yang Zhang, Yuan Li

**Affiliations:** Medical Center for Human Reproduction, Beijing Chao-Yang Hospital, Capital Medical University, Beijing, China

**Keywords:** GnRH agonist, GnRH antagonist, ovarian stimulation, LH levels, reproductive outcomes

## Abstract

**Objective:**

To verify if patients with deep ovarian suppression following gonadotropin releasing hormone (GnRH) agonist long protocol may benefit from a modified GnRH antagonist protocol based on luteinizing hormone (LH) levels.

**Design:**

Retrospective cohort study.

**Setting:**

University-based hospital.

**Patients:**

110 patients exhibited ultra-low LH levels during ovarian stimulation using GnRH agonist long protocol.

**Intervention(s):**

As all the embryos in the first cycle were exhausted without being pregnant, these patients proposed to undergo a second cycle of ovarian stimulation. 74 of them were treated with a modified GnRH antagonist protocol based on LH levels. Other 36 patients were still stimulated following GnRH agonist long protocol.

**Main Outcome Measure:**

The primary outcome was live birth rate (LBR). The second outcomes were biochemical pregnancy rate, clinical pregnancy rate (CPR), ongoing pregnancy rate (OPR) and cancellation rate.

**Results:**

Reproductive outcomes were much better in the modified GnRH antagonist protocol. The OPR and LBR were much higher in the GnRH antagonist protocol group than in the GnRH agonist long protocol group [odds ratio (OR) 3.82, 95% confidence interval (CI) 1.47, 10.61, P=0.018; OR 4.33, 95% CI 1.38, 13.60, P=0.008; respectively]. Meanwhile, the cancellation rate was much lower in the GnRH antagonist protocol group (OR 0.13, 95% CI 0.02, 0.72; P=0.014). Mean LH level during stimulation did not have a predictive value on live birth. However, it was independently associated with the occurrence of ongoing pregnancy (OR 2.70, 95% CI 1.25, 5.85; P=0.01). The results of sensitivity analyses were consistent with the data mentioned above. The patients got completely different and excellent clinical outcomes in their second cycles stimulated with the modified GnRH antagonist protocol.

**Conclusion:**

Patients with deep ovarian suppression following GnRH agonist long protocol may benefit from a modified GnRH antagonist protocol based on LH levels.

## Introduction

Luteinizing hormone (LH) plays an important role in promoting steroidogenesis, folliculogenesis, oocyte maturation, ovulation, formation and maintenance of corpus luteum (1, 2). Basic and clinical evidences have indicated that a threshold of LH stimulation is required for adequate follicular development and oocyte maturation (3–5). Ultra-high or low level of LH would do harm to pregnancy outcomes (6, 7). However, the threshold of LH has remained to be controversial till now. Actually, serum LH level during ovarian stimulation was not equivalent to LH activity. In one hand, like follicle stimulating hormone receptor (FSHR), polymorphisms in the LHR genes could impact ovarian response to stimulation and *in vitro* fertilization (IVF) outcomes (8, 9). In the other hand, the genetic variant type of the beta-subunit of LH (v-β LH), which had a shorten half-life period and low activity, was significantly widespread in different ethnical groups. Women with this mutation exhibited hypo-sensitivity to exogenous FSH and often showed amenorrhea and infertility (10, 11).

Gonadotrophin releasing hormone (GnRH) agonist long protocol has been widely used in the past few decades. Endogenous LH secretion was profoundly suppressed (<;1.2IU/L, severe LH deficiency) following GnRH agonist in some patients (2). For some of them, low LH level may have no detrimental effect on oocyte development and quality of the embryos. As it is known that < 1% of LHR be occupied could provoke a best steroidogenic response (12). While for others, the laboratory and clinical outcomes were very poor.

GnRH antagonist protocol has been increasingly used for ovarian stimulation. The use of GnRH antagonist during the late follicular phase can effectively prevent the occurrence of a premature LH surge (13, 14). As ovarian stimulation was performed without pituitary down regulation, LH might maintain a relatively high level. Meanwhile, neither traditional fixed nor flexible GnRH antagonist protocol was recommended for these patients with severe LH deficiency, in which, the administration of antagonist might also lead to lower LH activity. In our previous study, we proposed a modified GnRH antagonist protocol based on LH levels (15). It could maintain LH activity to a maximal level while avoiding a premature LH surge. The present study aims to verify if patients with deep ovarian suppression following GnRH agonist long protocol may benefit from this protocol.

## Materials and Methods

### Patients

110 infertile women were recruited from the Medical Center for Human Reproduction, Beijing Chao-Yang Hospital, Capital Medical University, from January 2016 to January 2018. Patients were included for analysis when they fulfilled the following criteria: 23–40 years old; normal ovarian reserve assessed on the third day of a spontaneous cycle (FSH<10 IU/L, 8<antral follicle count (AFC) <20) (16–18); showing deep ovarian suppression following GnRH agonist long protocol [LH<1.2 IU/L on the initial day of stimulation, according to the defining concentration for the diagnosis for World Health Organization (WHO) type I anovulation (The European Recombinant Human LH Study Group, 1998)] in the first IVF/intracytoplasmic sperm injection (ICSI) cycles; all the embryos been exhausted with no live birth. The exclusion criteria included the following: body mass index (BMI)>28 kg/m^2^; diagnosis of a congenital or acquired uterine abnormality (such as a uterine malformation, adenomyosis, submucous myoma, or intrauterine adhesion); autoimmune, thyroid and chromosomal abnormalities; the presence of only one ovary. All patients involved in the present study provided an informed consent. The study was approved by the Ethics Committee of Beijing Chao-Yang Hospital, Capital Medical University. Analyses of data was performed in accordance with the rules and regulation with approvals from the ethics committee of our hospital.

### Study Design

The present study is a retrospective, single-center cohort study. As all the embryos have been exhausted with no live birth, the 110 patients accepted a second IVF/ICSI cycle. It is not appropriate to place most of the blame on the ovarian stimulation protocol as the cycle and laboratory parameters are acceptable (data not shown in the text). So, 74 patients were treated with a modified GnRH antagonist protocol based on LH levels. And other 36 patients were still stimulated using GnRH agonist long protocol.

### Ovarian Stimulation

A modified GnRH antagonist protocol based on LH levels was performed for 74 patients as mentioned before with some adjustments (15). Briefly, 150-300 IU r-FSH (Gonal F, Merck Serono, Germany) was administered daily from Day 2 or Day 3 of the menstrual cycle. The initial r-FSH dose was decided according to the patient’s age, AFC and BMI, and this remained fixed for four to five days. Then gonadotropin dosage might be adjusted according to hormone levels and follicle development. Hormone analyses were performed four to five times during stimulation, as follows: ① day 1 of stimulation; ② 4–5 days after stimulation initiation; ③ 2 days later, i.e., 6–7 days after initiation; ④ the day of triggering. Additionally, morning urine LH level testing was done from day 6 of stimulation on the day with no serum hormone test, by the patient themselves. If a positive result was observed, blood was taken for LH measurement immediately.

Considering that these patients showed deep ovarian suppression following GnRH agonist long protocol, administration of antagonist might lead to an even lower LH activity. So the traditional GnRH antagonist protocol was adjusted in the study, with administration and the dosage of antagonist based on LH levels from day 6 of ovarian stimulation. Based on our clinical experience, no antagonist was administered if LH level was lower than 4 IU/L. If 4≤LH levels<6 IU/L, 0.125mg cetrorelix acetate (Cetrotide R; Merck) was given daily for 2 days until the next blood test. If 6≤LH levels<10 IU/L, 0.25mg cetrorelix acetate was given daily for 2 days. If 10≤LH levels<15 IU/L, 0.375mg cetrorelix acetate was given daily for 1 day. If LH levels≥15 IU/L, 0.5mg cetrorelix acetate was given daily for 1 day. The decision to continue antagonist co-treatment was based on subsequent LH results >4 IU/L until trigger day.

Other 36 patients were still stimulated using GnRH agonist long protocol. Down- regulation was initiated on Day 21 of the previous cycle, using Triptorelin 0.05mg (Decapepty, Ferring, Sweden) daily for at least 14 days. Following down regulation, if serum E_2_<50 pg/mL, LH<5 IU/L and no follicle with diameter larger than 8 mm by vaginal ultrasound scanning, an individualized dose of 150-300 IU r-FSH was administrated daily. Gonadotropin dosage might also be adjusted according to hormone levels and follicle development.

For both protocols, r-LH was supplemented 75 or 150 IU/day if the LH level was very low or the development of follicles was inappropriate (<3 follicles of ≥8 mm on Day 6 of stimulation). The r-FSH dose could be titrated based on physician judgment. When more than two follicles were ≥18 mm in diameter, 250 μg of recombinant human chorionic gonadotropin (rhCG, Ovidrel, Merck Serono, Germany) was administered to induce final oocyte maturation. Ovum pick up (OPU) was performed 36 h after administration of rhCG. Retrieved oocytes were fertilized by either IVF or ICSI depending on sperm quality.

### Embryo Transfer and Luteal Phase Support

Cleavage embryos on Day 3 after OPU were graded by morphological criteria on the basis of the number and size of blastomere and the percentage of fragmentation (19). No more than three embryos were transferred. The rest embryos were cultured for two or three more days, and good quality blastocysts were vitrified. The blastocyst score was assessed according to Gardner morphological criteria, on the basis of the degree of expansion and the development of the inner cell mass and trophectoderm (20). Luteal phase support was provided with vaginal progesterone gel (Crinone, Merck Serono, Germany) 90mg/day and dydrogesterone (Duphaston, Abbott Laboratories, IL, USA) 20 mg/day. If pregnancy was achieved, luteal phase support was continued until 12 weeks’ gestation.

Fresh embryo transfer was cancelled if patients had risk of ovarian hyperstimulation syndrome (OHSS), an unfavorable endometrium (endometrial thickness of ≤6 mm or ≥16 mm, fluid in cavity or endometrial polyp), progesterone level≥1.5 ng/ml on the day of hCG trigger, or no embryo. For frozen embryo transfer, the endometrium was prepared either with a natural cycle regimen or an artificial cycle regimen, based on the decision of doctors.

### Outcomes and Measures

The primary outcome was live birth rate (LBR). The second outcomes were biochemical pregnancy rate, clinical pregnancy rate (CPR), ongoing pregnancy rate (OPR), and cancellation rate. A β-hCG level above 10 IU/L was defined as a positive biochemical pregnancy. Clinical pregnancy was diagnosed when the ultrasound revealed a gestation sac and fetal heartbeat after 2-3 weeks from the positive HCG test. Ongoing pregnancy was defined as pregnancy progressing beyond 12 weeks after OPU. Cancellation rate was defined as the number of cycles with no embryo for transfer divided by the number of OPU cycles.

### Blood Samples and Hormone Assays

Serum hormone concentrations were measured using a competitive chemiluminescence immunoassay using commercial kits obtained from Roche Diagnostics. Serum FSH, LH and E2 levels were routinely tested on the second or third day of menstruation before IVF/ICSI treatment, and after pituitary suppression by GnRH agonist injection. During ovarian stimulation, serum E_2_, LH and progesterone were measured regularly, mostly between 4 and 5 times, until the day of rhCG administration. Blood test was performed at a relatively fixed time to minimize the possible influence of circadian rhythm changes on hormone levels (8 a.m. to half past 8 a.m.).

All measurements were performed according to the manufacturer’s instructions.

### Statistical Analysis

Continuous data were expressed as means ± SD unless otherwise stated. An independent sample t-test was used for continuous variables that were normally distributed, and the Mann–Whitney U-test was used for data not normally distributed. Categorical data were represented as frequency and percentage; differences in these variables were assessed by chi-square test or Fisher’s exact test. LBR per woman was assessed both crudely and using multivariable logistic regression analysis. A two-sided alpha of 5% was applied in the univariate analysis. The variables were assessed for colinearity before added in the final model. The decision to add each measured potential confounder in the model was based on previous scientific evidence and the results in the unadjusted analyses, in which, confounders were selected on the basis of their associations with LBR or a change in effect estimate of more than 10%. The model for CP, OP and LB included the following variables: follicular output rate (FORT, calculated as the ratio between the number of pre-ovulatory follicles obtained in response to FSH administration and the pre-existing pool of small antral follicles.), no. of oocyte, no. of good quality embryos, no. of embryos transferred, age, BMI, Gn dosage, LH on trigger day, P on trigger day, mean LH level during stimulation, GnRH antagonist dosage, and rLH dosage. Hosmer-Lemshow test was applied to validate the discriminatory power and accuracy of the multivariate regression model. A higher value for the Hosmer-Lemshow test indicated better model fit.

We also did sensitivity analyses for the outcomes of the 74 patients who were treated with GnRH agonist long protocol in the first cycle and GnRH antagonist protocol in the second cycle.

Statistical analysis was performed using the Statistical Package for the Social Sciences (SPSS, version 22.0). All statistical tests were two sided. A P-value <0.05 was considered statistically significant.

## Results

**Figure 1:**
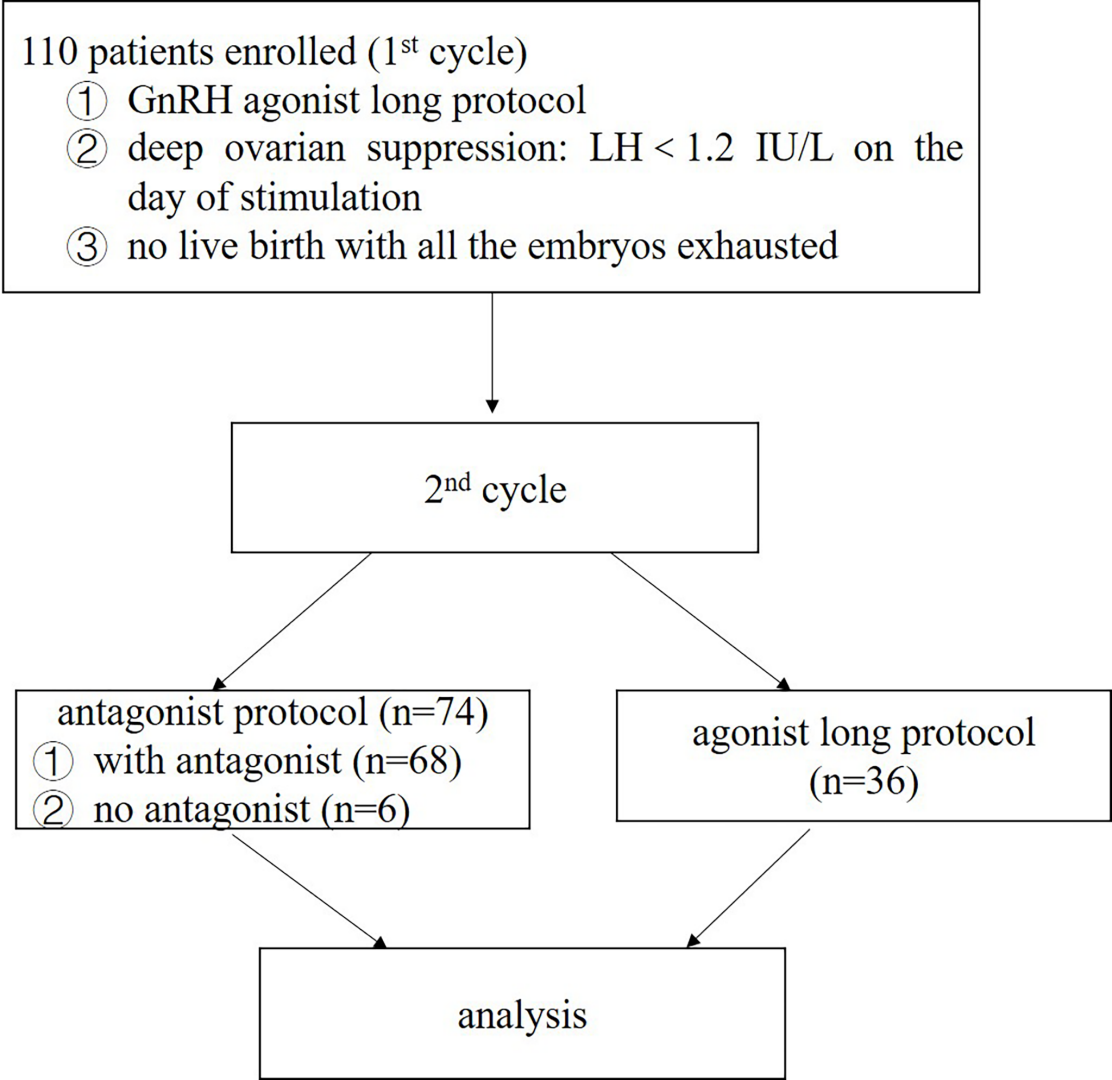
Flowchart of patient flow.

**Figure 1** displayed the flowchart of patient flow. Over a 2-year period, 110 patients were included in this study. Baseline patient characteristics and demographic data were comparable between the two protocol groups (**Table 1**). In the present study, only two patients had LH reached 10 IU/L during ovarian stimulation. So, adjusting the antagonist’s doses according to the LH levels when LH became > 10 IU/L was not common. As the effect of the antagonist was dose-dependent, the level of LH could easily decrease to a safe range after 1 day of cetrorelix acetate of 0.375mg. No cycle was cancelled due to premature ovulation. 68 (91.90%, 68/74) patients received a relatively small dose of antagonist based on LH levels during ovarian stimulation (LH > 4 IU/L) and the other 6 (8.10%, 6/74) patients did not receive any antagonist.

**Table 1:** Baseline patient characteristics and demographic data.

	antagonist protocol	agonist long protocol	P value
N	74	36	
Age (years)	34.1 ± 3.5	32.7 ± 4.9	0.16
BMI (kg/m^2^)	22.7 ± 3.1	23.3 ± 3.3	0.68
Basal FSH (IU/L)	6.9 ± 2.5	7.0 ± 1.9	0.61
Basal LH (IU/L)	4.1 ± 1.7	4.5 ± 1.9	0.55
Basal E_2_ (pg/ml)	46.5 ± 19.7	59.5 ± 25.9	0.09
AFC	13.2 ± 4.5	13.0 ± 4.6	0.71
Duration of infertility (years)	3.6 ± 2.3	4.2 ± 3.9	0.84
Indications			
Male factor	18 (24.3%)	9 (25.0%)	
Tubal factor	41 (55.4%)	20 (55.5%)	
Combined factors	11 (14.9%)	6 (16.7%)	
Unexplained infertility	3 (4.1%)	1 (2.8%)	
Other	1 (1.3%)	0	

Data presented as means ± SD or n (%); BMI, body mass index; AFC, antral follicle count.

Many ovarian stimulation characteristics were similar in the two treatment groups, whereas FORT, P and LH levels on day of trigger, and no. of oocytes were much higher in antagonist protocol group. rLH supplementation was much more in GnRH agonist long protocol group to keep LH to a certain level and maintain normal follicle development. During ovarian stimulation, hormones including LH were tested 4-5 times, representing early, mid, and late follicular phases. Then mean LH level could be figured out, which was much higher in the GnRH antagonist protocol group (2.84 ± 1.19 *vs.* 1.51 ± 0.34 IU/L, P<0.0001, **Table 2**).

**Table 2:** Cycle parameters in antagonist *vs.* agonist long protocol group.

	antagonist protocol (n= 74)	agonist long protocol (n = 36)	P value
FORT (%)	0.86 ± 0.35	0.68 ± 0.31	0.01
Gn dosage (IU)	2375.27 ± 641.80	2712.50 ± 1145.80	0.11
Gn duration (days)	10.00 ± 1.48	10.56 ± 1.70	0.10
E_2_ on day of trigger (pg/ml)	3498.52 ± 1895.19	3044.39 ± 1344.10	0.15
P on day of trigger (ng/ml)	0.97 ± 0.58	0.71 ± 0.34	0.005
LH on day of trigger (IU/L)	2.70 ± 2.31	1.83 ± 0.81	0.005
Mean LH level during stimulation (IU/L)	2.84 ± 1.19	1.51 ± 0.34	<0.0001
rLH dosage (IU)	295.50 ± 344.25	787.50 ± 370.56	<0.0001
Em thickness (mm)	10.27 ± 1.98	10.78 ± 2.47	0.28
No. of oocytes	13.32 ± 6.06	11.00 ± 4.55	0.02
No. of 2PN	8.22 ± 4.41	7.33 ± 3.99	0.29
No. of good quality embryos	3.78 ± 2.82	3.11 ± 3.02	0.26
No. of embryos transferred	1.70 ± 0.93	1.39 ± 0.84	0.08

FORT, follicular output rate.

### Reproductive Outcomes

26 (35.14%) of 74 women in the GnRH antagonist protocol group had live birth, which was much higher than in the GnRH agonist long protocol group (4 of 36 [11.11%]; OR 4.33, 95% CI 1.38,13.60; P=0.008, **Table 3**). Analyses of the secondary outcomes showed that biochemical pregnancy rate, CPR, and OPR were significantly higher in the antagonist protocol group as compared with the GnRH agonist long protocol group, too (**Table 3**). 2 (2.70%, 2/74) and 6 (16.67%, 6/36) embryo transfer cycles were cancelled because of failed embryo development in the two groups, respectively.

**Table 3:** Clinical outcomes in antagonist *vs.* agonist long protocol group.

	antagonist protocol	agonist long protocol	OR (95%CI)	P value
Biochemical pregnancy	38/74 (51.35)	8/36 (22.22)	3.69 (1.48,9.16)	0.004
Clinical pregnancy	28/74 (37.84)	6/36 (16.67)	3.04 (1.12,8.22)	0.024
Ongoing pregnancy	27/74 (36.49)	4/36 (11.11)	3.82 (1.47,10.61)	0.018
Live birth	26/74 (35.14)	4/36 (11.11)	4.33 (1.38,13.60)	0.008
Cancellation	2/74 (2.70)	6/36 (16.67)	0.13 (0.02,0.72)	0.014

Data presented as n/total (%). Statistical analysis was carried out using Chi-squared test.

OR, odds ratio; CI, confidence interval.

**Table 4** summarized the results of the multivariate regression analyses of the CPR, OPR and LBR in the GnRH antagonist protocol arm. The results showed that the number of embryos transferred was associated with increased odds for clinical pregnancy, ongoing pregnancy and live birth. GnRH antagonist dosage and rLH supplementation dosage were not independent predictors for live birth (OR 1.52, 95% CI 0.68, 4.11, P=0.17; OR 2.20, 95% CI 0.59, 4.92; P=0.18; respectively). Mean LH level during stimulation did not have a predictive value on either clinical pregnancy or live birth (OR 1.73, 95% CI 0.93, 3.21, P=0.08; OR 1.71, 95% CI 0.97,3.26; P=0.08; respectively). However, it was independently associated with the occurrence of ongoing pregnancy (OR 2.70, 95% CI 1.25, 5.85; P=0.01). The association between LH level on trigger day and LBR was not statistically significant (OR 0.87, 95% CI 0.64,1.20; P=0.39). Similar results were shown for CPR and OPR (OR 0.99, 95% CI 0.72,1.38, P=0.99; OR 0.89, 95% CI 0.64,1.27, P=0.55; respectively). All the Hosmer-Lemeshow tests for the final model showed P > 0.05, which meant a good fit of the multivariate regression model.

**Table 4:** Multivariate regression results for reproductive outcomes: patients in the GnRH antagonist protocol arm.

Dependent variable	Independent variable	Adjusted OR (95%CI)	P value
Clinical pregnancy	FORT	0.24 (0.03, 2.03)	0.19
	no. of oocyte	1.29 (1.11,1.49)	0.001
	no. of good quality embryos	0.92 (0.74,1.14)	0.46
	no. of embryos transferred	2.68 (1.36, 5.28)	0.01
	age	0.92 (0.81,1.05)	0.21
	BMI	1.00 (0.84,1.19)	0.97
	Gn dosage	1.00 (0.99,1.00)	0.51
	LH on trigger day	0.99 (0.72, 1.38)	0.99
	P on trigger day	1.46 (0.41, 5.04)	0.56
	mean LH level during stimulation	1.73 (0.93, 3.21)	0.08
	GnRH antagonist dosage	1.47 (0.48, 3.74)	0.20
	rLH dosage	1.26 (0.53, 4.18)	0.15
Ongoing pregnancy	FORT	0.32 (0.03, 3.06)	0.32
	no. of oocyte	1.26 (1.08,1.47)	0.003
	no. of good quality embryos	1.04 (0.82,1.31)	0.76
	no. of embryos transferred	2.98 (1.43,6.20)	0.003
	age	0.91 (0.78,1.05)	0.20
	BMI	1.09 (0.90,1.32)	0.39
	Gn dosage	1.00 (0.99,1.00)	0.79
	LH on trigger day	0.89 (0.64,1.27)	0.55
	P on trigger day	2.21 (0.57, 5.54)	0.25
	mean LH level during stimulation	2.70 (1.25, 5.85)	0.01
	GnRH antagonist dosage	1.81 (0.49, 3.62)	0.19
	rLH dosage	1.47 (0.82, 2.79)	0.23
Live birth	FORT	0.28 (0.04, 1.89)	0.19
	no. of oocyte	1.09 (0.97,1.22)	0.16
	no. of good quality embryos	1.18 (0.97,1.44)	0.10
	no. of embryos transferred	2.37 (1.26,4.44)	0.01
	age	0.97 (0.86,1.09)	0.58
	BMI	1.00 (0.85,1.17)	0.96
	Gn dosage	1.00 (0.99,1.00)	0.85
	LH on trigger day	0.87 (0.64,1.20)	0.39
	P on trigger day	1.68 (0.51, 5.48)	0.39
	mean LH level during stimulation	1.71 (0.97,3.26)	0.08
	GnRH antagonist dosage	1.52 (0.68, 4.11)	0.17
	rLH dosage	2.20 (0.59, 4.92)	0.18

A two-sided alpha of 5% was applied in the univariate analyses. The variables were assessed for colinearity before added in the final model. The stepwise method was used to fit the best model.

### Sensitivity Analyses

The results of sensitivity analyses were consistent with the data mentioned above. Gn duration was shorter and E_2_ on day of trigger was lower in the second cycle (using antagonist protocol). LH on day of trigger and during ovarian stimulation were much higher in the second cycle (2.71 ± 2.31 *vs.* 1.84 ± 0.66 IU/L, P=0.003 and 2.84 ± 1.19 *vs.* 1.43 ± 0.39 IU/L, P<0.0001, respectively). Whereas more good quality embryos were obtained in the second cycle stimulated with antagonist protocol (**Supplementary Table 1**). Reproductive outcomes were very poor for these patients in the first cycle, with 6 cycles (8.11%) cancelled because of no embryo obtained and only 2 patients (2.70%) reached ongoing pregnancies. However, none of them got live birth. In the second cycle, the same patients got completely different and excellent clinical outcomes. 26 of them got live birth (35.14%, OR 19.5, 95% CI 4.42,85.99, P=0.000, **Supplementary Table 2**).

## Discussion

LH is essential for normal folliculogenesis and oocyte maturation. Ultra-high or low level of LH would do harm to pregnancy outcomes (6, 7). Till now, there is still no consensus definition of LH threshold for adequate folliculogenesis and steroidogenesis. Previous studies indicated that the lower limit of the LH threshold ranged between 0.5 and 1.2 IU/L (6, 7). It is known that hypogonadotropic amenorrhea is also defined as LH < 1.2 IU/L. Taken together, LH<1.2 IU/L on the initial day of stimulation is used as the definition of deep ovarian suppression following GnRH agonist long protocol in the present study. Previous studies showed that low LH levels on the day of GnRH-a trigger were associated with a low mature oocyte yield and a suboptimal response to GnRH agonist trigger (21, 22). Another study demonstrated that low serum LH levels on the day of GnRH-a trigger was associated with reduced ongoing pregnancy and live birth rates (23). Some studies with hCG trigger have also reported that a late follicular phase LH threshold exists below which adverse effects on the reproductive outcomes will occur (7, 24, 25). On the contrary, no association between endogenous LH level and pregnancy outcome has been reported in other studies (26–28).

Previous studies have demonstrated LHR expression in small follicles of 6–8mm in diameter (29). The results supported the evidence that LH played an important role even from the early stage of follicular growth and during the whole phase. We should not merely pay attention to the late phase of follicle development. Some researchers evaluated the effect of early- and mid-follicular LH concentrations on the ovarian response and pregnancy outcomes. Humaidan P et al. demonstrated that circulating levels of LH on day 8 had a significant impact on ovarian response and pregnancy outcome (6). Lahoud R et al. showed that low mid-follicular levels of LH had a significant impact on ovarian response but not on live birth rates. A fall in LH level of ≥50% from the early- to mid-follicular phase resulted in a lower live birth rate (7). Actually, neither LH level on day 8 or mid follicular LH level, nor LH level on trigger day could represent the LH level during whole ovarian stimulation and follicle development. Multivariate regression results of the present study showed that there was a positive correlation between mean LH level during stimulation and ongoing pregnancy.

Because of polymorphism in the LHR gene and v-β LH, LH requirement during ovarian stimulation might vary from patients. Our studies demonstrated that those patients with severe LH deficiency following pituitary down regulation may have special genetic background. Some gene variants or single nucleotide polymorphisms (SNPs) may be related to LH deficiency (unpublished data). For some patients, the low level of LH following pituitary down regulation might be enough, but for others, it was definitely not, which could result in insufficient oocyte development, and even had detrimental effect on embryo quality and clinical outcomes.

There were, broadly speaking, two ways of increasing LH activity. One way was supplementation of LH. Till now, LH supplementation in ovarian stimulation remained a controversial issue. Fa’bregues et al. believed that LH supplementation did not increase ovarian response and implantation rates in patients of older reproductive age stimulated with GnRH agonist protocol (30). Whereas other previous studies demonstrated that supplementation with rLH seemed to benefit treatment outcome (31–34). In the present study, rLH was supplemented in both protocol groups to increase LH activity and maintain normal follicular development. The results showed that much less rLH was supplemented during ovarian stimulation in GnRH antagonist protocol group than agonist long protocol (295.50 ± 344.25 *vs.* 787.50 ± 370.56 IU, P<0.0001). It meant better reproductive outcomes with less cost in antagonist protocol group. To further compare the ovarian response and clinical outcomes between the two protocols, we carried out a sensitivity analysis in those patients underwent both the two protocols. The data also indicated better clinical outcomes for GnRH antagonist protocol with less Gn duration, less rLH supplementation and more good quality embryos. These patients, with no live birth following all the embryos exhausted in the first cycle (stimulated with GnRH agonist long protocol), got a live birth rate up to 35.14% in the second IVF cycle. Results of the multivariate regression analyses in the GnRH antagonist protocol arm showed that rLH supplementation dosage was not independent predictor for live birth. However, r-LH supplementation based on the clinicians’ judgment may be a source of bias in the present study.

Another way to protect LH activity was to keep LH to a relatively high concentration by adjusting stimulation protocol. Pituitary down regulation should be avoided and GnRH antagonist protocol was recommended in those patients with deep ovarian suppression. GnRH antagonist directly and rapidly inhibited gonadotrophin release within several hours through competitive binding to pituitary receptors. This property allowed their flexible use, almost at any time during follicular phase. Several different regimens have been described including multiple‐dose fixed, multiple‐dose flexible, and single‐dose protocols (35, 36). We seldom performed a daily dosage of GnRH antagonist over 0.5 mg in clinical practice. Further, based on our previous data, women with LH levels lower than 4 IU/L did not require addition of antagonists for LH suppression. Administration of antagonist would further decrease LH levels and have detrimental effect to reproductive outcomes. Antagonist administration only when LH level was over 4 IU/L would not increase cycle cancellation rate because of unexpected LH surge (15). We believed it was important to be clear that antagonist should not be supplemented mechanically, as in traditional flexible (antagonist administration based on the estrogen level or the size of follicles) or fixed antagonist protocol (antagonist administration based on the stimulation days). Some patients may much more “sensitive” to antagonist priming, with “overdose” of which may cause endogenous LH levels to decrease excessively and poor clinical outcomes. They may have special genetic background, such as some gene variants or SNPs (our unpublished data). However, those special patients could not be distinguished before ovarian stimulation through baseline characteristics. Thus, we suggested GnRH antagonist be supplemented according to LH levels and follicular development through ovarian stimulation. This protocol was LH based flexible GnRH antagonist protocol, as shown previously (15) and modified in the present study. The results showed a better ovarian response (higher FORT), less cost (less rLH supplementation and lower Gn dosage) and better clinical outcomes.

With each embryo transfer cycle, there is a certain probability of a live birth. So, each additional IVF cycle increases the cumulative live birth rate as long as there are available embryos for transfer. This study demonstrated that live birth rates in the patients treated with the modified GnRH antagonist protocol in the second IVF cycle were higher than those treated with agonist protocol (35.14% *vs* 11.11%). This improvement was to a large extent related to the adjustment of ovarian stimulation protocol and the addition of rLH, especially for the “special” patients showing deep ovarian suppression following GnRH agonist downregulation.

An increase in serum P level may be observed at the end of ovarian stimulation. It was associated with low serum LH concentrations and high serum FSH concentrations (37). Previous study reported that serum P concentrations at the time of hCG administration were lower with GnRH antagonist as compared with GnRH agonist protocols (38). Our results were different from the previous study. In the present study, both LH and P levels on hCG trigger day were higher in the GnRH antagonist protocol group (2.70 ± 2.31 *vs.* 1.83 ± 0.81 IU/L, 0.97 ± 0.58 *vs.* 0.71 ± 0.34 ng/ml, respectively). It may at least partially because we did not use antagonist daily from day 6 to trigger day in the LH based GnRH antagonist protocol group. In the sensitivity analysis, LH levels on trigger day were also much higher in the 2^nd^ cycle (GnRH antagonist protocol). However, the results of the multivariate regression analysis did not show positive relationship between P levels on hCG trigger day and LBR (OR 1.68, 95% CI 0.51, 5.48, P=0.39; **Table 4**). And in the sensitivity analysis, there was no significant difference in P level on the trigger day between the two cycles (0.97 ± 0.58 *vs.* 1.09 ± 0.50 ng/ml, P=0.17; **Supplementary Table 1**). It may because of the limited sample size of the present study. The consequences of serum P increase on cycle outcome remain to be controversial. A meta-analysis concluded that P elevation did not have an adverse effect on pregnancy rate (39). However, a previous study including more than 4,000 cycles has reported a deleterious effect of serum P values higher than 1.5 ng/mL (38). Further large-scale study was needed to corroborate.

The present study had some limitations on account of its retrospective nature and small sample size. It may not power to prevent statistical detection of further laboratory and/or clinically significant differences. Although the multivariate regression controlled for confounders, there could still be residual variables which are not considered. Secondly, the influence of circadian rhythm changes on LH levels cannot be completely avoided although hormone test was performed at a relatively fixed time. A serum LH of 4 IU/L as the cut off value to decide whether or not GnRH antagonist is based on our own clinical experience. To some extent, it may not be the best discriminatory value and the LH assays currently used do not always accurately reflect the LH bioactivity. LHR polymorphism and other gene variants could not be totally avoided by the modified GnRH antagonist protocol, which may be also a source of bias. Thirdly, the present study failed to get a cutoff value or threshold of mean LH level during ovarian stimulation that be optimal for successful reproductive outcomes. Fourthly, the findings of the current study cannot be extrapolated to single fresh blastocyst stage transfer, which is the current mode of modern practice. Further prospective randomized trials are necessary to verify the availability of this LH based flexible GnRH antagonist protocol.

## Conclusion

In conclusion, patients with deep ovarian suppression following GnRH agonist long protocol may benefit from a modified GnRH antagonist protocol based on LH levels. Further prospective randomized trials are necessary to verify the availability of this protocol.

## Data Availability Statement

The original contributions presented in the study are included in the article/**Supplementary Material**. Further inquiries can be directed to the corresponding author.

## Ethics Statement

The studies involving human participants were reviewed and approved by Institutional Review Board of Beijing Chao-Yang Hospital, Capital Medical University. The patients/participants provided their written informed consent to participate in this study.

## Author Contributions

SL: data collection and statistical analysis; drafting of the manuscript. ML, LL, HLi, DQ, HR, HS, and YZ: patient’s treatment and revising of the manuscript. YL: supervision of the study concept and review of manuscript. All authors contributed to the article and approved the submitted version.

## Funding

This study was supported by 1351 Talent training Program of Beijing Chao-Yang Hospital (CYXX-2017-20, CYMY-2017-21); Capital Health Development Scientific Research Project (Independent Innovation, 2020-1-2039); Beijing Health Promotion Foundation (2019-09-05); 2018 Fertility Research Program of Young and Middle-aged Physicians-China Health Promotion Foundation; 2020 Fertility Research Program of Young and Middle-aged Physicians-China Health Promotion Foundation; Beijing Hospitals Authority Youth Programme (QML20200301).

## Conflict of Interest

The authors declare that the research was conducted in the absence of any commercial or financial relationships that could be construed as a potential conflict of interest.
