# Supplementary material for: Patients With Deep Ovarian Suppression Following GnRH Agonist Long Protocol May Benefit From a Modified GnRH Antagonist Protocol: A Retrospective Cohort Study
Source: Front Endocrinol (Lausanne). 2021 Jul 13;12:618580. doi: 10.3389/fendo.2021.618580 (PMC8314175; doi:10.3389/fendo.2021.618580)
Supplement: Supplementary file 1 [file Table_1.docx]

Supplementary Table 1 Sensitivity analyses between two cycles within the patients stimulated with different protocols (self-control)

|  | 2^nd^ cycle | 1^st^ cycle | P value |
| --- | --- | --- | --- |
| protocol | antagonist protocol | agonist long protocol |  |
| n | 74 | 74 |  |
| FORT (%) | 0.86±0.35 | 0.85±0.36 | 0.82 |
| Gn dosage (IU) | 2375.27±641.80 | 2448.65±585.50 | 0.46 |
| Gn duration (days) | 10.00±1.48 | 10.49±1.20 | 0.03 |
| E_2_ on day of trigger (pg/ml) | 3498.52±1895.19 | 4155.98±2049.60 | 0.05 |
| P on day of trigger (ng/ml) | 0.97±0.58 | 1.09±0.50 | 0.17 |
| LH on day of trigger (IU/L) | 2.71±2.31 | 1.84±0.66 | 0.003 |
| Mean LH level during stimulation (IU/L) | 2.84±1.19 | 1.43±0.39 | <0.0001 |
| rLH dosage (IU) | 295.50±344.25 | 500.25±345.75 | 0.001 |
| Em thickness (mm) | 10.27±1.98 | 10.12±1.77 | 0.63 |
| No. of oocytes | 13.32±6.06 | 12.70±6.43 | 0.54 |
| No. of 2PN | 8.22±4.40 | 7.27±4.61 | 0.20 |
| No. of good quality embryos | 3.78±2.82 | 2.68±2.40 | 0.01 |
| No. of embryos transferred | 1.70±0.93 | 1.43±0.95 | 0.08 |
