# Supplementary material for: Patients With Deep Ovarian Suppression Following GnRH Agonist Long Protocol May Benefit From a Modified GnRH Antagonist Protocol: A Retrospective Cohort Study
Source: Front Endocrinol (Lausanne). 2021 Jul 13;12:618580. doi: 10.3389/fendo.2021.618580 (PMC8314175; doi:10.3389/fendo.2021.618580)
Supplement: Supplementary file 2 [file Table_2.docx]

Supplementary Table 2 Sensitivity analyses for reproductive outcomes between two cycles (self-control)

|  | antagonist protocol  (2^nd^ cycle) | agonist long protocol  (1^st^ cycle) | OR（95%CI） | P value |
| --- | --- | --- | --- | --- |
| Biochemical pregnancy | 38/74 (51.35) | 10/74 (13.51) | 6.75 (3.01,15.14) | 0.00 |
| Clinical pregnancy | 28/74 (37.84) | 4/74 (5.41) | 10.65 (3.50,32.37) | 0.00 |
| Ongoing pregnancy | 26/74 (35.14) | 2/74 (2.70) | 19.50 (4.42,85.99) | 0.00 |
| Live birth | 26/74 (35.14) | 0/74 | - | - |
| Cancellation | 2/74 (2.70) | 6/74 (8.11) | 0.31 (0.06,1.61) | 0.27 |

Data presented as n/total (%). Statistical analysis was carried out using Chi-squared test.
